# PhenoFam-gene set enrichment analysis through protein structural information

**DOI:** 10.1186/1471-2105-11-254

**Published:** 2010-05-17

**Authors:** Maciej Paszkowski-Rogacz, Mikolaj Slabicki, M Teresa Pisabarro, Frank Buchholz

**Affiliations:** 1 Max Planck Institute of Molecular Cell Biology and Genetics, Pfotenhauerstr. 108, 01307 Dresden, Germany; 2 Structural Bioinformatics, BIOTEC TU Dresden, Tatzberg 47-51, 01307 Dresden, Germany

## Abstract

**Background:**

With the current technological advances in high-throughput biology, the necessity to develop tools that help to analyse the massive amount of data being generated is evident. A powerful method of inspecting large-scale data sets is gene set enrichment analysis (GSEA) and investigation of protein structural features can guide determining the function of individual genes. However, a convenient tool that combines these two features to aid in high-throughput data analysis has not been developed yet. In order to fill this niche, we developed the user-friendly, web-based application, PhenoFam.

**Results:**

PhenoFam performs gene set enrichment analysis by employing structural and functional information on families of protein domains as annotation terms. Our tool is designed to analyse complete sets of results from quantitative high-throughput studies (gene expression microarrays, functional RNAi screens, *etc*.) without prior pre-filtering or hits-selection steps. PhenoFam utilizes Ensembl databases to link a list of user-provided identifiers with protein features from the InterPro database, and assesses whether results associated with individual domains differ significantly from the overall population. To demonstrate the utility of PhenoFam we analysed a genome-wide RNA interference screen and discovered a novel function of plexins containing the cytoplasmic RasGAP domain. Furthermore, a PhenoFam analysis of breast cancer gene expression profiles revealed a link between breast carcinoma and altered expression of PX domain containing proteins.

**Conclusions:**

PhenoFam provides a user-friendly, easily accessible web interface to perform GSEA based on high-throughput data sets and structural-functional protein information, and therefore aids in functional annotation of genes.

## Background

Analysis of large sets of results derived from high-throughput experiments is a challenging but promising field of study. Enrichment analysis is a very powerful strategy helping researchers in identifying biological processes or pathways related to their studies. Most of the currently available tools (*i.e. *Onto-Express [1], DAVID [2], FatiGO+ [3], ConceptGene [4] and others reviewed in [5]) search for enrichment of Gene Ontology (GO) terms [6], KEGG pathways [7] or other functional properties in a pre-selected subset of genes by contrasting it with the background set, usually a whole genome. This approach strongly relies on a chosen hit selection algorithm and user-defined thresholds. Moreover, the experimental results (*i.e. *level of expression or phenotype strength) are not considered. There are few applications overcoming these limitations by performing gene set enrichment analysis (GSEA) [8]. They search for gene annotations enriched on the top or the bottom of a complete list of genes ranked by their experimental values. This allows even mild effects to contribute to the overall enrichment score. However, to our knowledge, annotations used by available GSEA tools have so far primarily been used in combination with GO terms, pathways or transcription factors, and only few of these applications are web-based (*e.g. *GSEA [9], FatiScan [3], GeneTrail [10]).

In recent years, access to high-resolution protein structural information has increased considerably. Many new structures reveal the presence of domains known from other proteins, and the domain composition of a protein can help forming a hypothesis about its biological function (*e.g. *a homeodomain fold indicates a transcription factor activity involved in cellular differentiation [11]). Moreover, Hahne *et al*. demonstrated, that the domain composition of proteins could be used for predicting their pathway membership [12]. There are many databases classifying and providing information about protein families, domains, regions and functionally relevant sites. InterPro [13] constitutes a repository that integrates a number of the most well established sources of data: PROSITE [14], HAMAP [15], Pfam [16], PRINTS [17], ProDom [18], SMART [19], TIGRFAMs [20], PIRSF [21], SUPERFAMILY [22], Gene3D [23] and PANTHER [24]. We have developed a GSEA web application that can be used for analysing data from large-scale experiments (phenotypes, gene expression, *etc*.). Our tool combines the experimental results with annotations from the databases integrated in InterPro (called 'member databases'), thereby allowing a streamlined structure/function annotation of proteins. Utilization of information about protein domain families in GSEA is a novel approach that can be used in parallel to other enrichment analysis applications.

## Implementation

### Data management

PhenoFam is a Java web application running on a Tomcat 5.5 server. It uses a MySQL database to store mappings between various protein, gene or probe names and identifiers related to member databases of InterPro. This database is an easily updatable compilation of the current releases of the Ensembl database [25]. Client-server communication is mainly handled by AJAX technologies. User-uploaded data sets and calculation results are stored as session objects on the server side for at least 30 minutes after closing the browser window.

### Identifiers association

**Figure 1:**
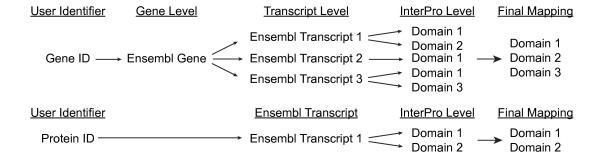
**Identifier mapping procedure**. Gene-related identifiers (*e.g. *Gene Names) are mapped to Ensembl Gene IDs and further to all protein-coding Ensembl Transcript IDs. Each of the transcripts is associated with protein features from the InterPro database. Redundant identifiers are removed in the final mapping. Protein- or transcript-related identifiers (*e.g. *UniProt IDs) are directly linked to Ensembl Transcript IDs and then to protein features.

One of the key features of our application is that it accepts as input a wide range of identifiers used in all genomes integrated in the Ensembl database [25]. Identifiers provided by the user are translated into respective Ensembl (gene, or transcript) identifiers and, using mappings from the InterPro database, linked to none, one or several protein domains or features from different InterPro database members (Figure 1). Reversing the mapping, each protein domain is linked with at least one user identifier and at least one experimental value.

It must be noted that all identifier mappings are based on contents of the Ensembl database, which establishes the links based on sequence similarity of entities stored in remote databases to sequences stored in Ensembl. This approach provides the highest quality of associations. However, care must be taken if gene-related identifiers are used. Due to alternative splicing, different gene products may be composed of different protein domains or even encode different proteins (*i.e. *shift in the reading frame). In such cases, a value associated with the user-provided identifier is mapped to all possible protein features that can be associated with the gene (Figure 1).

### Gene set enrichment analysis

To test if a set of values associated with a given domain is significantly higher or lower than the remaining set of values, we use the Mann-Whitney *U*-test. The *U*-test is the most powerful nonparametric alternative to the Student's *t*-test. Its main advantage is that it makes no assumptions about the underlying distributions and is more robust in case of outliers. The *U*-test is also implemented in other popular GSEA tools, *i.e. *GeneTrail [10] or PANTHER [26,27]. Other applications (*i.e. *GOdist [28], GSEA [9]) implemented the Kolmogorov-Smirnov (KS) test, another non-parametric procedure that checks whether two samples (values associated with a given domain and the other values) may be assumed to come from the same distribution. However, the KS test is also sensitive to differences in the general shapes of the distributions, which limits its use for our PhenoFam application. Parametric analysis, which was proposed by Kim *et al*. and implemented in PAGE [29], is also not suitable for GSEA of protein domains because many domains are associated with small number of proteins (< 10). In those cases, the normality criteria required for parametric tests might not be satisfied. Adjustment for multiple testing is done using the false discovery rate (FDR) control procedure designed by Benjamini and Hochberg [30] and resulting *q*-values are obtained by applying Storey's algorithm [31,32]. Additionally, we calculate a Herrnstein's *ρ *statistic [33], which is an unbiased measure of the overlap between distributions of values in the two compared sets. It can reach values between 0 and 1, where 0.5 indicates a complete overlap of the two distributions and both extreme values show a complete separation. This statistic shows how much a median of domain-associated values differs from a median of the other values, and together with the *p*-value can help identifying domains of interest. We recommend using it for sorting results that passed the significance-threshold criteria. Due to the fact that InterPro is a collection of partially redundant databases, the enrichment analysis and the adjustment for multiple testing procedure are performed for each database independently. Otherwise, treating InterPro as a uniform set of annotations would lead to a significant underestimation of the results.

### User interface

To implement the user interface and to ensure compatibility with all major browsers, we used the Google Web Toolkit (GWT) framework. We have designed a simple and user-friendly data management system for storing uploaded data sets and the analysis results. It allows users to investigate and compare multiple data sets at the same time.

**Figure 2:**
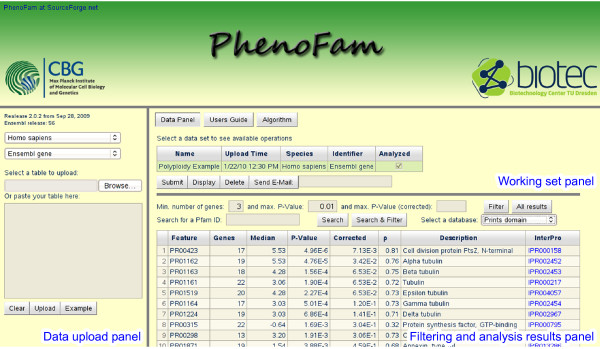
**PhenoFam web interface**. The main user interface display is divided into three panels. The 'Data upload panel' allows uploading data sets for the GSEA analysis either by pasting the data or by selecting a text file. All uploaded data sets are displayed in the 'Working set panel', where the user can submit data for the analysis, view the results in the browser or send them by e-mail. The sortable table with results is displayed in the 'Filtering and analysis results panel'. The top section of the panel contains a form that provides searching and filtering functionality. The displayed table contains a list of significantly enriched PRINTS domains.

Our GSEA algorithm reports the following information: a member database identifier, the domain description, a number of user identifiers associated with the domain, a median of the values, a *p*-value reported by the Mann-Whitney *U*-test, a FDR corrected *p*-value, a *ρ *statistic and the InterPro identifier. The results associated with one of the selected InterPro member databases are displayed in a pageable table (Figure 2) that can be sorted and filtered. We also provide a possibility to search for specific domains. For each selected domain, we also show a table of associated values together with original identifiers, UniProt accessions and descriptions. A brief user's guide to PhenoFam is provided in Additional file 1, as well as on the application web site.

## Results

PhenoFam allows many data sets as the starting point, such as results of microarray studies, systematic RNA interference (RNAi) screens, ChIP-Chip/ChIP-Seq experiments or comparative mass-spectrometry (*i.e. *SILAC) results. To test the utility of PhenoFam, we analysed a data-set derived from a genome-scale cell cycle progression RNAi screen [34]. In this screen, a genome-wide study of genes was carried out providing *z*-scores for cell cycle progression phenotypes (*i.e. *cells in G1, S, G2/M phases and polyploidy) for each knockdown.

**Figure 3:**
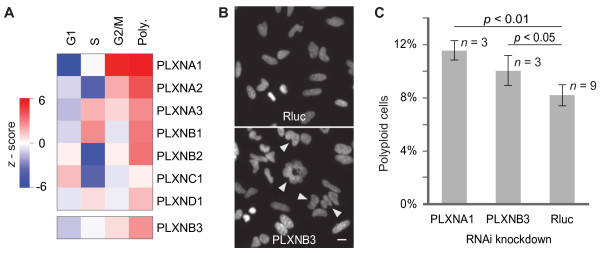
**Plexins are enriched for polyploidy RNAi phenotypes**. **A**. Cell cycle phenotypic profiles after knockdown of plexins. The top heatmap shows data extracted from the genome-wide RNAi screen [34], and the bottom heatmap shows the cell cycle profile after knock-down of PLXNB3. The PLXNB3 profile was obtained from the automated analysis of microscopy images, which quantifies proportions of cells in different phases of the cell cycle. *z*-scores were calculated by normalization to the mean and standard deviation of respective values obtained from the analysis of negative control images. **B**. Fluorescence microscopy images of HeLa cells 48 hours after transfection of esiRNA (endoribonuclease-prepared siRNA) against Rluc (negative control) and PLXNB3. The images show DAPI-stained nuclei, and arrows indicate cells with polyploidy phenotype. The scale bar represents 10 μm. Investigation of both images shows that the knock-down of PLXNB3 results in a polyploidy phenotype compared to the negative control condition. **C**. Quantification of the image analysis of polyploidy phenotypes among cells treated by different silencing triggers (≈ 5 000 cells per replicate). Error bars represent one SD. Student's *t*-test confirmed that the ratio of polyploid cells is significantly increased after knock-down of indicated plexins, compared to the control condition (Rluc).

A PhenoFam analysis of the complete RNAi data-set revealed that plexins containing a cytoplasmic RasGAP domain were enriched (*p *< 0.005) for polyploidy phenotypes (Figure 3A, Table 1). Knockdown of most transcripts encoding these genes resulted in an increase of polyploidy cells. Although in the published RNAi screen [34] only genes with the strongest polyploidy phenotypes of *z*-score > 6 were selected for further investigation, the PhenoFam analysis suggests that plexins not passing this criteria might also have a function in cytokinesis.

**Table 1:** Normalized values of polyploidy RNAi phenotypes of plexins, from the primary cell cycle progression screen.

Ensembl ID	Gene	Polyploidy (*z*-score)
ENSG00000114554	PLXNA1	13.15
ENSG00000076356	PLXNA2	4.05
ENSG00000130827	PLXNA3	2.72
ENSG00000164050	PLXNB1	3.14
ENSG00000196576	PLXNB2	3.33
ENSG00000004399	PLXND1	1.45
ENSG00000136040	PLXNC1	0.32

Moreover, based on this result we predicted that knockdown of the gene PLXNB3, which belongs to the same family, but had not been tested in the screen, would also increase the degree of polyploidy. Indeed, an increased number of polyploid cells were measured after PLXNB3 knockdown (Figure 3), indicating that depletion of this gene, like other plexins with cytoplasmic RasGAP domains, influences proper cytokinesis. This example demonstrates that PhenoFam can be a valuable support for selecting hits from the RNAi screens.

To show that PhenoFam is also suitable for analysis of other large-scale data-sets, we examined publicly available gene expression data that compares transcriptomes of human breast carcinoma and healthy tissue [35]. GSEA of this data-set using GeneTrail [10] showed that genes whose expression is altered in breast cancer are significantly enriched with the 'signal transduction' and 'cell differentiation' gene ontologies, highliting the importance of these biological processes during cellular transformation (data not shown). However, the analysis with GeneTrail did not provide information of enrichment of certain protein domains. In contrast, analysis of the same data-set with PhenoFam showed that among differentially expressed genes, Ras-family proteins and phox (PX) domain-containing proteins were enriched (*p *< 0.001, data not shown).

Ras GTPases are known to play a role in breast cancer development [36] and, therefore, it is not surprising that this group of proteins was enriched in this set. Proteins containing a PX domain are involved in cell signalling, vesicular trafficking, protein sorting and lipid modification, and are primarily found in sorting nexins [37]. Previous studies suggest that various sorting nexins are involved in leukemia [38], colon tumorigenesis [39] and, in general, contribute to cell cycle progression in mammalian cells [40]. However, their role in breast cancer has not been described so far. Our PhenoFam anaysis suggests that proteins with PX domains are frequently misregulated in breast cancer. Hence, we propose that these proteins should be investigated for a possible role in breast cancer development.

## Conclusions

PhenoFam is a computational tool designed to analyse experimental results by integration of functional and structural information about protein families. The distinct features of our application include a user-friendly interface and a broad range of supported genomes and identifiers. It should also be noted that our algorithm, in contrast to existing software, treats the InterPro repository as a collection of partially redundant databases, which improves the power of our testing procedure. Using a specific example, we show that the application can be used as an additional hit selection tool for functional screens. Typical hit selection procedures (*i.e. z*-score or quantile-based normalization) apply thresholds that can be passed only by genes showing the strongest phenotypes, which often leads to a high false-negatives rate. In case of our GSEA method, a domain may appear to be significantly enriched despite moderate phenotypes of the associated genes. From the potential relationship between the domain and the investigated biological process, genes with moderate phenotypic scores are considered in the list of hits selected from the screen, thereby reducing the false-negative rate.

We also demonstrated that PhenoFam can help forming novel hypothesis based on gene expression data. Accordingly, PhenoFam should be useful in analysing results of other high-throughput experiments, such as ChiP-Chip/ChiP-Seq and comparative mass-spectrometry. In summary, together with other enrichment analysis tools, PhenoFam can assist in annotating genes of unknown function and in discovering new functions of already characterised genes.

## Availability and requirements

**Project name**: PhenoFam

**Project homepage**: http://www.phenofam.org/

**Operating system(s)**: Platform independent (web-based application)

**Programming language**: Java

**Other requirements**: A web browser with JavaScript support

**License**: GNU GPLv3 http://www.gnu.org/licenses/gpl-3.0.html

**Any restrictions to use by non-academics**: None

## Authors' contributions

MPR implemented the application and drafted the manuscript. MS carried out the experiments and contributed in writing the manuscript. MTP contributed in designing the application and in writing the manuscript. FB coordinated the study and contributed in writing the manuscript. All authors read and approved the final manuscript.

## Supplementary Material

Additional file 1 **PhenoFam User's Guide**. The file contains a PDF version of the User's Guide provided on the PhenoFam home page. Click here for file
