# Supplementary material for: PhenoFam-gene set enrichment analysis through protein structural information
Source: BMC Bioinformatics. 2010 May 17;11:254. doi: 10.1186/1471-2105-11-254 (PMC2881086; doi:10.1186/1471-2105-11-254)
Supplement: Additional file 1 — PhenoFam User's Guide. The file contains a PDF version of the User's Guide provided on the PhenoFam home page. [file 1471-2105-11-254-S1.PDF]

# PhenoFam

## http://www.phenofam.org/

## Users Guide

I. Select the correct species and identifier type.

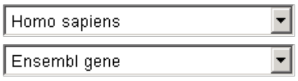


II. Browse for a file with the data or paste the table in the box on the left. your table should contain two columns separated by either spaces or tabs. The first column should contain identifiers of a chosen type and the second should contain values. Your data should look like this:

```
ENSG00000033800 0.26
ENSG00000130254 0.48
ENSG00000153187 -1.76
...
```

The more data you provide, the more accurate results you get.

III. Press the upload button and wait until your data is imported. You can upload several lists and access them later on.

IV. After successful upload the main panel should appear and you should see a page similar to this:

Select a data set to see available operations

| Name | Upload Time | Species | Identifier | Analyzed |
|---|---|---|---|---|
| PhenoFamTestSet.csv | 3/2/09 6:15 PM | Homo sapiens | Ensembl gene | ☐ |

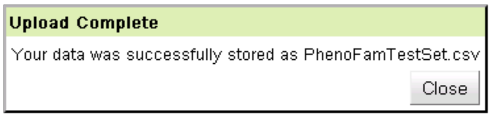


Here you can see a message box informing of a successful upload and behind there is a table of the data sets that were uploaded in a current session.

V. Close the message box and select your data set. A list of available operations should appear below the table:

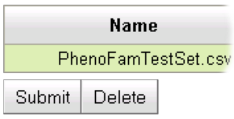


VI. In order to start calculation press the *Submit* button. The time of calculation depends on your sample size. If you would like to have the results send you by e-mail, simply type your address and press the button:

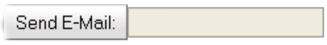


VII. When the calculations are finished the results are automatically displayed below:

| | Feature | Genes | Median | P-Value | Corrected | ρ | Description | InterPro |
|---|---|---|---|---|---|---|---|---|
| 1 | PF03953 | 19 | 5.53 | 4.76E-5 | 1.56E-1 | 0.76 | Tubulin/FtsZ, C-terminal\|Tubulin/FtsZ, 2-layer sandwich domain | IPR008280, IPR018316 |
| 2 | PF00091 | 20 | 4.28 | 2.27E-4 | 3.74E-1 | 0.73 | Tubulin/FtsZ, GTPase domain | IPR003008 |
| 3 | PF00004 | 40 | -0.74 | 7.78E-4 | 4.08E-1 | 0.36 | ATPase, AAA-type, core | IPR003959 |
| 4 | PF00481 | 17 | 1.87 | 1.13E-3 | 4.08E-1 | 0.71 | Protein phosphatase 2C, N-terminal\|Protein phosphatase 2C-related | IPR014045, IPR001932 |
| 5 | PF01545 | 8 | -1.22 | 1.32E-3 | 4.08E-1 | 0.19 | Cation efflux protein | IPR002524 |

The table contains the following columns:

- Feature - identifier from the InterPro member database
- Genes - number of user uploaded IDs associated with the feature
- Median - median of values associated with the feature
- P-Value - p-value associated with the Mann-Whitney U test
- Corrected - false discovery rate (FDR) corrected p-value (Benjamini-Hochberg)
- ρ - Herrnstein's ρ statistic
- Description - description of the feature
- InterPro - InterPro identifiers associated with the feature

You can sort the table by clicking on column headers. If the list is too long then just the first page is displayed and the page navigation arrows 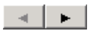 appear below the table.

VIII. Above the results table you can see a set of text boxes and buttons allowing you to filter and search the result set:

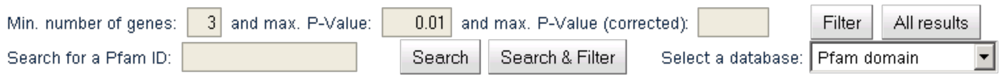


- Filter - display only results matching all the three criteria (if provided)
- All results - opens a new window containing full result set, can be easily copied and pasted to another application
- Search - searches for specified text in Pfam IDs and descriptions
- Search & Filter - just like the Search button but also filters the results according to the provided criteria
- Select a database - displays results calculated for the selected InterPro member database (see InterPro User Manual)

IX. By clicking on a row of the results table you can display original values that were associated with the Pfam ID. A new table will appear:

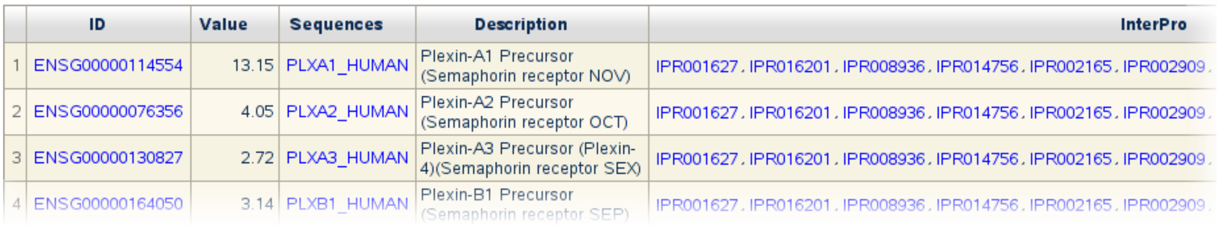

| | ID | Value | Sequences | Description | InterPro |
|---|---|---|---|---|---|
| 1 | ENSG00000114554 | 13.15 | PLXA1_HUMAN | Plexin-A1 Precursor (Semaphorin receptor NOV) | IPR001627 , IPR016201 , IPR008936 , IPR014756 , IPR002165 , IPR002909 , |
| 2 | ENSG00000076356 | 4.05 | PLXA2_HUMAN | Plexin-A2 Precursor (Semaphorin receptor OCT) | IPR001627 , IPR016201 , IPR008936 , IPR014756 , IPR002165 , IPR002909 , |
| 3 | ENSG00000130827 | 2.72 | PLXA3_HUMAN | Plexin-A3 Precursor (Plexin-4)(Semaphorin receptor SEX) | IPR001627 , IPR016201 , IPR008936 , IPR014756 , IPR002165 , IPR002909 , |
| 4 | ENSG00000164050 | 3.14 | PLXB1_HUMAN | Plexin-B1 Precursor (Semaphorin receptor SEP) | IPR001627 , IPR016201 , IPR008936 , IPR014756 , IPR002165 , IPR002909 , |

The table contains the following columns:

- ID - user provided identifier. In some cases a link to the original database is provided (i.e. Ensembl gene id).
- Value - the original value
- Sequences - a list of UniProt IDs linked to the provided identifier
- Description - descriptions of the ID
- InterPro - a list of InterPro identifiers associated with the ID
